# Quantifying disease severity in amanitin-intoxicated patients by fusing common laboratory values

**DOI:** 10.3389/ftox.2026.1995921

**Published:** 2026-10-14

**Authors:** Philipp L. S. Ohland, Stephan Halle, Korbinian Brand, André Bleich, Steven R. Talbot

**Affiliations:** 1 Institute for Laboratory Animal Science, Hannover Medical School, Hanover, Germany; 2 Institute of Immunology, Hannover Medical School, Hanover, Germany; 3 Institute of Clinical Chemistry and Laboratory Medicine, Hannover Medical School, Hanover, Germany; 4 Institute of Medical Statistics, RWTH Aachen University Hospital, Aachen, Germany

**Keywords:** acute liver failure, Amanita phalloides, amatoxin poisoning, composite severity score, laboratory biomarkers, patient vital status

## Abstract

**Introduction:**

Amanitin intoxication following ingestion of amatoxin-containing mushrooms can cause acute hepatic and renal failure. We evaluated the feasibility of using an adapted Patient Vital Status (PVS) fusion score derived from routine laboratory parameters to assess disease severity in amanitin-intoxicated patients.

**Methods:**

In this retrospective single-center pilot study (2023–2024), pseudonymized laboratory data from 16 adults with urine-confirmed amanitin intoxication and 12 control patients with comparable laboratory measurements were analyzed. PVS was calculated using prothrombin time (Quick), alanine aminotransferase (ALT), aspartate aminotransferase (AST), bilirubin, and lactate dehydrogenase (LDH), with predefined normal values and an intermediate-care reference group (n = 5) for severity scaling.

**Results:**

Intoxicated patients had significantly higher maximum and mean PVS values than controls (PVSmax: 0.75 ± 0.54 vs. 0.12 ± 0.04; PVSmean: 0.32 ± 0.25 vs. 0.05 ± 0.02; mean ± standard deviation; both p < 0.001). Exploratory k-means clustering (k = 4) yielded four descriptive severity groups. The most severely affected patient reached a maximum PVS of 1.91 (ALT 10,373 U/L; AST 8,776 U/L; Quick 26.7%). Among patients with a matched ingestion time, the median time to peak PVS was 3.03 days (Q1–Q3, 2.13–3.54; n = 15). PVS was positively associated with Model for End-Stage Liver Disease (MELD) scores (p < 0.001). One patient died (last PVS 1.34). Urinary amanitin concentrations were not significantly associated with severity metrics.

**Discussion:**

These findings support the feasibility of adapting PVS for longitudinal laboratory-based severity assessment in amanitin intoxication. Given the exploratory nature and limited sample size of this pilot study, validation in larger prospective cohorts is warranted.

## 1 Introduction

Intoxication following ingestion of Amanitin-containing mushrooms can present severe clinical challenges, such as acute hepatic and renal failure (Broussard et al., 2001; Tan et al., 2022; Visser et al., 2024). The primary toxins responsible for these severe effects are α-, β-, and γ-Amanitin, found in several members of the Amanitaceae family of mushroom-forming fungi, including *Amanita phalloides*, commonly known as the death cap mushroom (Garcia et al., 2015; Letschert et al., 2006; Rodrigues et al., 2020). Amatoxins inhibit RNA polymerase II, which transcribes DNA into messenger RNA, thereby disrupting protein synthesis and causing subsequent cell death, primarily affecting the liver and kidneys (Magdalan et al., 2011a; Santi et al., 2012; Ye and Liu, 2018). Ingestion of *Amanita phalloides* can result in severe poisoning, which is characterized by gastrointestinal distress, hepatic and renal dysfunction, and potentially death. The toxin is highly stable *in vivo*, and continued enterohepatic circulation is a great concern in patients where intoxication is not suspected and treated early after Amanitin ingestion (Thiel et al., 2011).

The current methods for managing Amanitin intoxication include the detection of Amanitin levels in urine and the assessment of liver enzymes, creatinine, and lactate dehydrogenase (LDH) for the severity assessment. Elevated levels of aspartate aminotransferase (AST), alanine aminotransferase (ALT), total serum bilirubin, and international normalized ratio (INR) are commonly used biomarkers for evaluating liver damage and intoxication severity (Caré et al., 2024; Ganzert et al., 2005; Tan et al., 2022).

The early detection of Amanitin intoxication is of paramount importance for the improvement of clinical outcomes (Caré et al., 2024; Robinson-Fuentes et al., 2008). Inspection and testing of ingested mushrooms, ideally unprocessed from the prepared meal, along with a detailed patient interview, are critical to the evaluation of Amanitin poisoning in the emergency department. In Germany, local poison centers coordinate the rapid identification of mushrooms by specialists. If intoxication is suspected, antidotal treatment is usually started immediately, with confirmation based on urinary Amanitin detection and liver enzyme monitoring.

The quantification of the disease severity of intoxicated patients may facilitate a more profound comprehension of disease progression, early detection, and severity characterization. This study introduces an adapted version of the Patient Vital Status (PVS) fusion score, which employs laboratory values pertinent to Amanitin poisoning instead of vital signs for longitudinal severity evaluation (Ohland et al., 2024). It provides a comprehensive composite score based on physiological data, which can objectively assess and compare severity levels in different models and time points. The customizability allows broad application opportunities by changing subjects or variables. This scoring system is based on the Relative Severity Assessment (RELSA) score, designed for laboratory mice but adapted for clinical use in humans (Talbot et al., 2022). The PVS score was initially employed to facilitate continuous monitoring of physiological data in septic critical care patients, with the first application in pediatric patients. The present study evaluates the feasibility of using this approach to summarize longitudinal laboratory abnormalities in Amanitin intoxication.

The practical clinical management of Amanitin intoxication involves the use of specific antidotes, such as silibinin, benzylpenicillin, and N-acetylcysteine (Caré et al., 2024; Montanini et al., 1999; Tan et al., 2022). These antidotes have been shown to have varying degrees of effectiveness in mitigating the hepatotoxic effects of Amanitin (Magdalan et al., 2011a). The mechanisms by which these antidotes act differ, including inhibiting oxidative stress and enhancing detoxification pathways. These mechanisms aim to protect liver cells from the toxic effects of Amanitin (Magdalan et al., 2011b). Novel therapeutic approaches, such as using fractionated plasma separation and adsorption systems, have also been reported as a potential therapeutic approach in reducing Amanitin levels in the blood and improving patient outcomes without needing liver transplantation (Bergis et al., 2012). Additionally, experimental studies have highlighted the potential of using resveratrol and other antioxidants to mitigate the oxidative damage caused by Amanitin in hepatocytes (Sahin et al., 2018).

The Model for End-Stage Liver Disease (MELD) score, which is commonly used to assess liver function in severe liver disease, was initially based on bilirubin, INR, and creatinine (Kamath et al., 2001). MELD was subsequently updated to MELD-Na by incorporating sodium levels (Biggins et al., 2006; Kim et al., 2008; Nagai et al., 2018). In the present study, however, the original MELD formulation was used as an exploratory comparator to PVS. The adapted PVS score extends severity assessment by integrating liver enzymes and LDH into a comprehensive composite score. Amanitin poisoning is rare, which limited the number of intoxicated patients available for this observational pilot study (Caré et al., 2024). These cases were used retrospectively to develop a novel PVS application with different objectives and variables from those previously used and to demonstrate the feasibility of fusing them into a severity score (Figure 1). The study further evaluated its feasibility for assessing acute and severe disease events such as Amanitin poisoning. In addition, the study examined the correlation between disease severity and urinary Amanitin levels. Our working hypothesis was that the laboratory-based PVS would be higher in intoxicated patients than in controls and would summarize within-patient changes in laboratory abnormalities. Analyses of clustering, MELD associations, and urinary Amanitin concentrations were exploratory.

**FIGURE 1:**
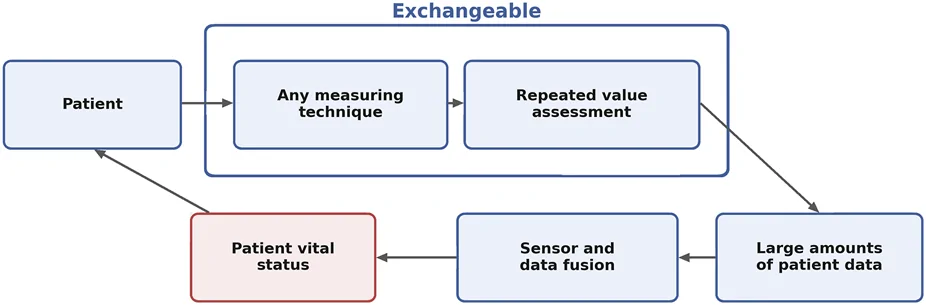
Fusion of Exchangeable Variables into a Comprehensive Severity Score. The schematic illustrates the general PVS workflow. Patient data can be acquired using exchangeable measuring techniques and repeated assessments, generating longitudinal data that are integrated by sensor and data fusion into the Patient Vital Status.

## 2 Materials and methods

### 2.1 Data collection

The Institute of Clinical Chemistry and Laboratory Medicine at the Hannover Medical School collected the pseudonymized raw data between 2023 and 2024 and included each laboratory variable measured. The data included information on the measuring device, the time of the request, a unique request number, and a unique patient identification number. The data were cleaned and compiled, resulting in a final set of variables. Each data entry was validated by clinicians before inclusion. The study was reviewed by the local Ethics Committee of Hannover Medical School, which raised no objections to the conduct of the study (reference no. 12557-BO-K-2026).

The intoxication group comprised adults with Amanitin intoxication confirmed by urinary Amanitin detection during 2023–2024. This was an opportunity sample of all available cases at Hannover Medical School meeting these criteria and providing sufficient routine laboratory measurements for the planned analyses. For time-course analyses, the recorded ingestion time was used. In the source data, ingestion times were classified as confirmed or estimated. A matched ingestion time was available for 15 of the 16 intoxicated patients; 12 were classified as estimated and 3 as confirmed. The control group comprised adult hospitalized patients from the same period with comparable laboratory measurements and without severe underlying internal disease; these were hospital controls rather than healthy volunteers. No additional diagnosis-specific exclusion criteria or care-setting restrictions were applied.

The final data set included 16 (57%) Amanitin-intoxicated patients and 12 (43%) control patients. All patients were adults. Systematic patient-level data on age, sex, time to admission, detailed level of care, treatments, renal replacement therapy, and comorbidities were not available in the pseudonymized dataset accessible for this analysis. Creatinine measurements were available as part of the routine laboratory data. One patient died. The laboratory variables analyzed were coagulation factors II and V, ammonia, prothrombin time (Quick), alanine aminotransferase (ALT), aspartate aminotransferase (AST), bilirubin, gamma-glutamyl transferase (GGT), creatinine, and lactate dehydrogenase (LDH). Urinary Amanitin concentrations were determined using the BÜHLMANN Amanitin ELISA (BÜHLMANN Laboratories AG) on a BEP III analyzer (Siemens) as part of routine toxicology diagnostics; the analytical limit of detection was 1.5 μg/L.

### 2.2 Score development and baseline definition

Disease quantification in this study is based on the Patient Vital Status, a previously published score for quantifying a patient’s multivariate vital status using continuously measured vital signs. Its initial proof of concept was applied to the vital signs of pediatric patients in the pediatric intensive care unit (PICU) using European Resuscitation Council normal values (Ohland et al., 2024; Van de Voorde et al., 2021). The PVS uses a custom set of variables and scales their deviations from normal relative to variable-specific reference deviations. These reference deviations are defined using a predefined reference population, resulting in a scaled severity weight (w). This technique relates each value at each time point. The final PVS score is calculated as the root mean square of all severity weights.

The predefined normal values used for PVS calculation corresponded to the clinical laboratory reference values available in the source data. Where reference values varied across periods and age/sex strata, the median reference value was used, resulting in Quick 100%, ALT 40 U/L, AST 20 U/L, bilirubin 10 μmol/L, and LDH 200 U/L (Supplementary Material S1). The 16 intoxicated patients had different disease courses. A subset of five intoxicated patients treated in an intermediate care unit (IMC) was selected as the intermediate-care reference group used to define the reference scaling. Accordingly, PVS = 1 corresponds to all contributing transformed deviations reaching their respective reference deviations.

For each laboratory variable j, the observed value xj was first normalized to its predefined normal value Nj as zj = 100 × xj/Nj. In the present implementation, all five laboratory variables were treated as ambivalent variables, meaning that deviations below 100% were reflected above 100% as z*j = 100 + |zj − 100|. The maximum transformed deviation observed in the five-patient reference set defined the variable-specific reference deviation Dj. The severity weight was therefore wj = (z*j − 100)/Dj. The Patient Vital Status at a given assessment was calculated as the root mean square of the m available severity weights: PVS = sqrt [(1/m) × Σj wj^2^]. Thus, PVS = 1 represents the internally defined reference scaling level and is not a validated clinical decision threshold.

### 2.3 Statistical analyses

All statistical analyses, including the adaptation of the PVS methodology, were performed using R (v4.2.1). The analysis was primarily conducted using the PVS package (available at https://github.com/phohland/PVS), with additional packages such as tidyverse, janitor, readxl, psych, rstatix, coin, lmerTest, and lsr for statistical computing. Data visualization was done using ggplot2, patchwork, and ggsignif.

Linear mixed-effects models (LMER) fit by restricted maximum likelihood (REML) with t-tests using Satterthwaite’s method, scaled predictors, and the patient ID as random effect were used to assess longitudinal associations between PVS, laboratory variables, time, and their interactions. These component-level models were used to describe longitudinal co-variation between PVS and its constituent laboratory variables and were not intended as independent validation of PVS. K-means clustering with k = 4 was used as an exploratory descriptive partition of patient-level maximum PVS values. The choice of four clusters was pragmatic, to provide descriptive granularity across the observed PVS range, and was not based on a formal silhouette, gap-statistic, or phenotype-discovery criterion. The Shapiro-Wilk test was utilized to assess normality. Welch’s t-test was used to compare the means of the two groups. Cohen’s d was calculated as an effect size. A linear regression model was used to analyze bivariate associations between Amanitin level and other variables. All hypothesis tests were two-sided, with an alpha level of 0.05. The significance levels for interpreting the resulting p-values were as follows: not significant (ns.), 0.05 (*), 0.01 (**), 0.001 (***), and 0.0001 (****).

For Quick, ALT, AST, bilirubin, and LDH, time from ingestion to the first observed measurement crossing the predefined PVS normal value in the clinically adverse direction and to the respective nadir or peak was summarized as median (Q1–Q3), using the same predefined normal values as in the PVS calculation (Quick 100%, ALT 40 U/L, AST 20 U/L, bilirubin 10 μmol/L, and LDH 200 U/L). As a sensitivity analysis, Quick was additionally scored one-sided. Quick values at or above 100% contributed no Quick-related deviation, whereas Quick values below 100% retained the original scaling relative to the Quick reference deviation; all other PVS components and reference scaling were unchanged.

For the longitudinal mixed models, the number of observations, fixed-effect estimates with standard errors and 95% confidence intervals, random-intercept variance, and residual variance are reported in Supplementary Material S4. Model assumptions were assessed by visual inspection of residual-versus-fitted and normal Q-Q plots.

The MELD score was calculated according to the original publication using INR values estimated from prothrombin time (Quick) with a predefined conversion scheme (Kamath et al., 2001). The predefined conversion was: Quick ≥ 100% = INR 1.0; 90 to <100% = 1.1; 80 to <90% = 1.2; 70 to <80% = 1.3; 60 to <70% = 1.4; 50 to <60% = 1.5; 40 to <50% = 1.7; 30 to <40% = 2.0; 20 to <30% = 2.5; and 10 to <20% = 3.5. Quick values < 10% yielded no estimated INR. Creatinine and bilirubin were converted from µmol/L to mg/dL; values below 1 mg/dL were set to 1, and INR values below 1 were set to 1. No dialysis adjustment or upper creatinine cap was applied. The resulting score was therefore treated as an approximate MELD comparator rather than a bedside-equivalent MELD calculation. Standardization was applied to the component-level mixed models described above. The separate mixed model evaluating the association between PVS and MELD used unstandardized PVS and MELD values.

### 2.4 Code availability

The PVS code used for score calculation and analysis is available on GitHub (https://github.com/phohland/PVS).

## 3 Results

### 3.1 PVS calculation and raw data analysis

The variables used for the PVS calculation were selected based on their clinical relevance for assessing Amanitin intoxication, particularly regarding the liver (Ganzert et al., 2005; Magdalan et al., 2011a). It was calculated using prothrombin time, ALT, AST, bilirubin, and LDH. The previously defined normal values and reference population were used.

The most severe prothrombin time, analyzed as Quick value, was 7% (typical reference range Quick value 80%–110%). The most severe ALT was 10,373 U/L, and AST was 8,776 U/L. This patient also achieved the highest LDH at 7,342 U/L and was in the ICU for 2 days. The highest achieved bilirubin was 265 μmol/L.

The patient with the highest ALT, AST, and LDH also achieved the highest PVS in the data set. At the time of the highest transaminases, the PVS was 1.91, also due to a Quick value of 26.7% and a bilirubin of 34 μmol/L. This patient reached this severe state 2.95 days after ingesting a poisonous mushroom. After this peak, the PVS dropped sharply by 0.98 and continued to decrease to 0.11 at the final available measurement. Figure 2 provides a visual representation of the progression of PVS in this patient and the corresponding variables.

**FIGURE 2:**
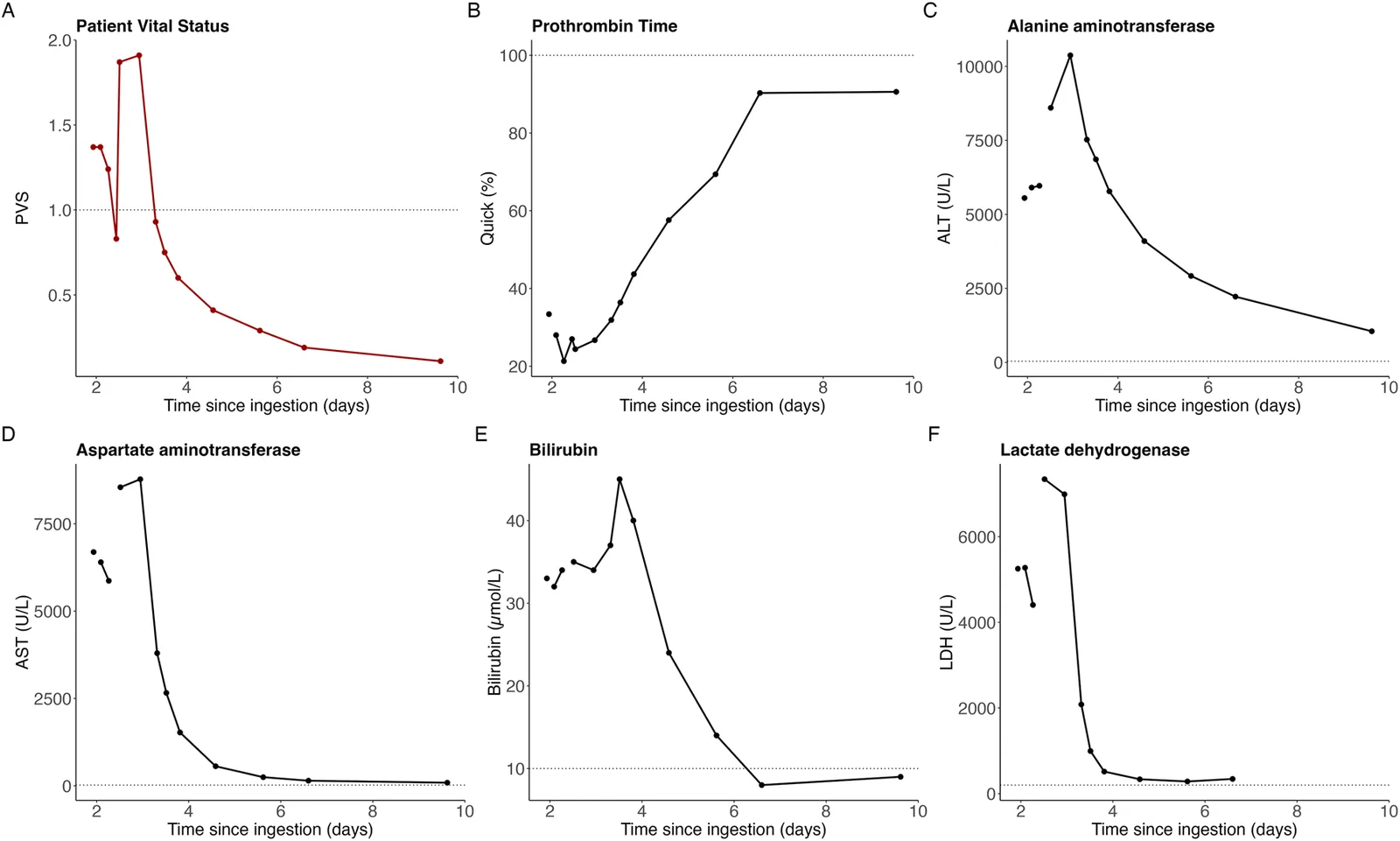
Visualization of PVS Progression and Corresponding Variables. The figure plots the PVS and its corresponding variables over time for the patient with the highest PVS, with time since ingestion in days on the x-axis. **(A)** The dotted horizontal line marks PVS = 1, the internally defined reference scaling level. PVS is on the y-axis. The first available PVS was 1.37 and, after a brief decline, increased to a maximum of 1.91. After this peak, the PVS showed a continuous decline and reached 0.11 at the final available measurement. The patient exceeded the reference scaling level of PVS = 1 and was therefore among the most severely affected patients in the cohort. **(B–F)** The horizontal dotted line represents the corresponding normal value, and the y-axis represents the variable associated with the corresponding unit. The figures illustrate the behavior of the variables throughout the measurement period. All variables showed a marked deviation from their typical values, affecting the PVS. If values for certain variables are missing, this is indicated by no line between the points.

Linear mixed-effects models assessed the relationship between PVS (dependent variable) and each laboratory measurement, time, and their interactions while accounting for patient-level random effects.

$$PVS_{ij}=\beta_{0}+\beta_{1}{\left(lab\;value\right)}_{ij}+\beta_{2}{\left(time\right)}_{ij}+\beta_{3}{\left(lab\;value\;*\;time\right)}_{ij}+b_{0i}+\epsilon_{ij}$$ (1)

The model can be expressed as shown in Equation 1, where β_0_ is the intercept, β_1_​, β_2_​, and β_3_​ represent the fixed effects for the laboratory values, time, and their interaction, b_0i_​ represents the random intercept for each patient i, and ϵ_ij_ is the residual error for observation j within patient i. Before analysis, all variables were standardized (scaled).

A decrease in prothrombin time was associated with increased PVS (df = 352.34, t = −18.74, p < 0.001). Increases in ALT (df = 151.62, t = 18.63, p < 0.001), AST (df = 451.92, t = 13.1, p < 0.001), bilirubin (df = 349.8, t = 7.4, p < 0.001) and LDH (df = 241.61, t = 7.16, p < 0.001) were also associated with increases in PVS. The effect of time was significant in the models for ALT (df = 58.3, t = −4.48, p < 0.001) and AST (df = 183.92, t = −3.42, p < 0.001) but not for prothrombin time (df = 57.71, t = 1.92, p = 0.059), bilirubin (df = 105.7, t = 0.03, p = 0.98) and LDH (df = 92.28, t = −0.94, p = 0.35). Significant interactions between laboratory values and time were observed for prothrombin time (df = 360.56, t = −4.58, p < 0.001) and ALT (df = 107.65, t = −3.72, p < 0.001), while the interactions for the other variables were not significant.

Residual diagnostics showed departures from ideal normality and constant residual variance, particularly in the distribution tails; accordingly, these component-level models are interpreted descriptively (Supplementary Material S4). Supplementary Material S2 presents descriptive statistics regarding the raw data in all intoxicated patients.

### 3.2 Severity clustering and group comparison

K-means clustering (k = 4) was used to partition patient-level maximum PVS values into four descriptive groups labeled low, mild, severe, and very severe. In the implemented analysis, clustering was performed on the combined patient-level dataset before the control patients were removed for presentation of the intoxicated cohort. Supplementary Material S3 shows the cluster assignments of the intoxicated patients.

Among the intoxicated patients, six were assigned to Cluster 1 (labeled low), four to Cluster 2 (mild), and three each to Clusters 3 and 4 (severe and very severe). The reported cluster centroids and within-cluster sums of squares refer to the combined clustering dataset from which the intoxicated-patient assignments were extracted. The centroids (cluster means) were 0.14 for Cluster 1, 0.78 for Cluster 2, 1.05 for Cluster 3, and 1.53 for Cluster 4. The within-cluster sum of squares was 0.07 for Cluster 1, 0.01 for Cluster 2, 0.02 for Cluster 3, and 0.21 for Cluster 4. For k = 4, the ratio of between-cluster to total sum of squares was 95.5%; this is reported as a descriptive clustering statistic and does not demonstrate four naturally occurring clinical phenotypes.

To assess differences in maximum and mean PVS between the intoxicated group (n = 16; PVS_max_: 0.75 ± 0.54; PVS_mean_: 0.32 ± 0.25) and the control group (n = 12; PVS_max_: 0.12 ± 0.04; PVS_mean_: 0.05 ± 0.02), Welch’s t-tests were performed; values are mean ± standard deviation. Both the highest PVS (df = 15.2, t = 4.63, p < 0.001, d = 1.64) and the mean PVS (df = 15.4, t = 4.33, p < 0.001, d = 1.53) were significantly higher in the intoxicated group than in the control group, and both comparisons showed large effect sizes (Figure 3).

**FIGURE 3:**
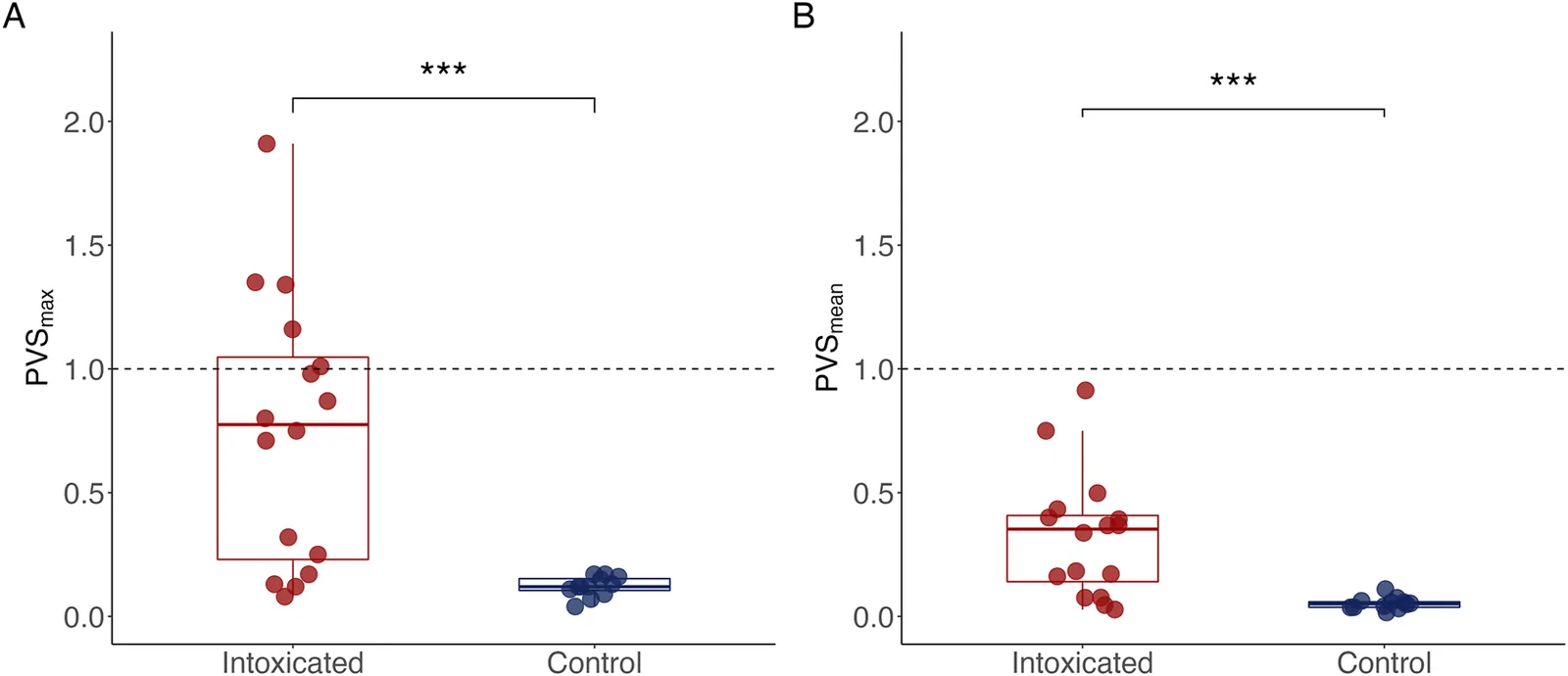
Comparison of PVS Between Intoxicated and Control Groups. Each figure contains the entire cohort of patients separated into the intoxicated (red) and control (blue) groups. Each point per plot represents a single patient. **(A)** The comparison of the maximum PVS of each patient with PVS_max_ is plotted on the y-axis. Several PVS values of intoxicated patients exceeded the reference scaling level of PVS = 1, while even the highest PVS_max_ values of the control group remained comparatively low. **(B)** The comparison of the mean PVS values. While the control group again remained low, there were intoxicated patients with average PVS scores up to 0.91. The intoxicated and control groups comprised 16 and 12 patients, respectively. Between-group comparisons used two-sided Welch’s t-tests (both p < 0.001; Cohen’s d = 1.64 for PVSmax and 1.53 for PVS_mean_). Boxes represent the interquartile range with the median as the central line; whiskers extend to 1.5 times the interquartile range, and points represent individual patients.

In the one-sided Quick sensitivity analysis, PVS_max_ remained higher in intoxicated than in control patients (0.73 ± 0.55 vs. 0.07 ± 0.05; p < 0.001; Cohen’s d = 1.70). PVS_mean_ likewise remained higher in intoxicated than in control patients (0.32 ± 0.26 vs. 0.03 ± 0.02; p < 0.001; Cohen’s d = 1.61) (Supplementary Material S6).

### 3.3 Outcome and amanitin analysis

PVS_last_ was evaluated descriptively within the intoxicated cohort (Figure 4). One of the 16 intoxicated patients died (PVS_last_ = 1.34). The mean PVS_last_ of the 15 surviving intoxicated patients was 0.10 ± 0.05. Because only one death occurred, no inferential comparison of mortality was performed. PVS_last_ was defined as the final laboratory-derived PVS available in the dataset for each patient; its timing was not standardized to a common clinical landmark such as discharge or death.

**FIGURE 4:**
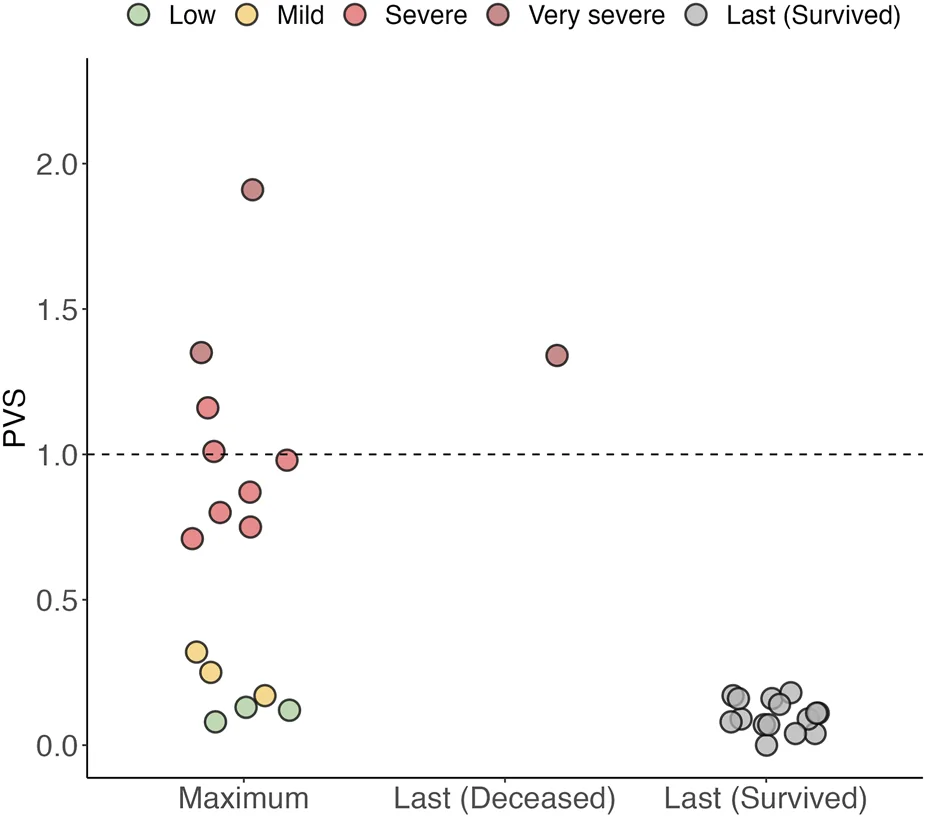
Descriptive PVS_last_ and Outcome Visualization in the Intoxicated Cohort. The analysis is restricted to the 16 intoxicated patients (15 survivors and one deceased patient). PVS_last_ denotes the final laboratory-derived PVS available in the dataset for each patient. Surviving patients are shown with their maximum and last available PVS; the deceased patient is shown at the last available PVS. Because only one death occurred, the figure is descriptive and no inference regarding mortality discrimination or prediction was performed. The dashed horizontal line marks PVS = 1, the internally defined reference scaling level.

Amanitin measurements were made at different times than the laboratory values, so several analyses were needed to explore the relationship between Amanitin levels and disease severity. A linear model of the Amanitin level measured at any time of stay and the corresponding highest PVS of an intoxicated patient showed no significant correlation (β = −0.0004, SE = 0.006, t = −0.07, p = 0.94). A linear model with Amanitin level and the nearest available PVS to the measurement time also found no significant relationship (β = 0.002, SE = 0.004, t = 0.61, p = 0.55). Finally, a linear model with Amanitin level and time since ingestion in days also found no significant association (β = −0.009, SE = 0.008, t = −1.12, p = 0.28; n = 15). Each model had the equation: Variable ∼ Amanitin Level. Figure 5 visualizes these relationships.

**FIGURE 5:**
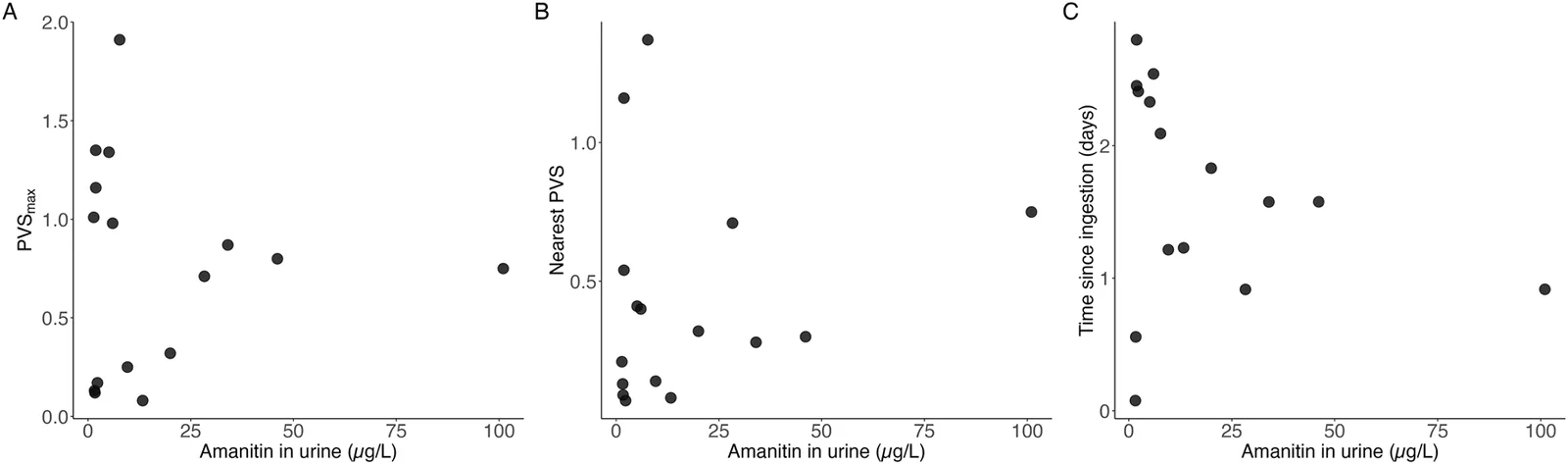
Association of Urinary Amanitin with PVS and Time Since Ingestion. Urinary Amanitin concentration is shown on the x-axis, consistent with the regression models in which Amanitin was the predictor. **(A)** PVS_max_. **(B)** Nearest available PVS to the Amanitin measurement. **(C)** Time since ingestion in days. No statistically significant association was detected in any model (**A**: p = 0.94, n = 16; **B**: p = 0.55, n = 16; **C**: p = 0.28, n = 15).

### 3.4 Patient Vital Status course after intoxication

A matched ingestion time was available for 15 of the 16 intoxicated patients. In these patients, the highest PVS occurred at a median of 3.03 days after ingestion (Q1–Q3, 2.13–3.54). The median time to the first observed abnormal measurement was 2.10 days (Q1–Q3, 1.96–2.67) for Quick, 2.31 days (2.00–2.70) for ALT, 2.07 days (1.93–2.48) for AST, 2.31 days (1.96–2.70) for bilirubin, and 2.05 days (1.92–2.64) for LDH. The corresponding median times to nadir or peak were 3.03 days (2.35–3.58) for Quick, 3.03 days (2.70–3.69) for ALT, 3.44 days (2.87–4.76) for AST, 4.44 days (3.59–7.50) for bilirubin, and 3.03 days (2.66–3.54) for LDH (Supplementary Material S5). Individual laboratory trajectories are also shown in Supplementary Material S5.

### 3.5 PVS compared to MELD

A linear mixed model was conducted to evaluate the association between PVS and MELD, with patient ID included as a random effect (equation: MELD ∼ PVS + (1 | patient ID)). An increase in PVS was associated with a significant rise in MELD (df = 210.03, t = 14.49, p < 0.001), with an estimate of 12.65, indicating that for every unit increase in PVS, MELD increases by approximately 12.65 units. Random effects showed a variance of 12.76 for the intercept across patients, with a residual variance of 9.93, indicating between-patient and within-patient variability.

## 4 Discussion

### 4.1 Quantifying disease severity with PVS

In this application, laboratory parameters replaced continuous vital signs, resulting in PVS calculation only during blood analysis. This reduced the number of PVS values but did not affect the generation of meaningful severity curves. Group comparisons showed significant results with high effect sizes for both mean and peak PVS. Due to the rarity of Amanitin poisoning, the study included a limited number of cases with a comparably sized control group for consistency. Exploratory k-means clustering partitioned maximum PVS values into four descriptive groups.

Associations between PVS and its constituent laboratory variables are expected by construction and should therefore be interpreted descriptively rather than as independent validation. This does not negate the purpose of the composite score, which is to integrate several laboratory abnormalities into a single multidimensional summary rather than to be statistically independent of its inputs.

Within the intoxicated cohort, the single fatal case had a markedly elevated PVS_last_ compared with surviving patients. This observation remains descriptive because only one death occurred and the timing of PVS_last_ was not standardized to a common clinical landmark. Severe clinical outcomes, including death and liver transplantation, occurred in patients with high PVS values, supporting the ability of PVS to summarize marked laboratory abnormalities over time, but not establishing prognostic discrimination.

Future discussions may explore alternative severity thresholds. Using a reference population allows for individualized severity scaling, but rigid criteria such as setting maximums at +100% of normal are impractical. Variables such as AST and ALT can be well outside normal ranges, while others, such as the Quick value, follow predefined scales. Detailed demographic and comorbidity data were unavailable; therefore, differences in age, sex, and underlying diseases could have contributed to the observed group differences and limited individualized reference-range adjustment. Because the reference scale was derived from only five patients within the intoxicated cohort, the absolute numerical PVS scale may be sensitive to the selected reference patients and requires external calibration in larger independent cohorts.

PVS was strongly associated with MELD. Because both scores share bilirubin and coagulation-related information, this association is best interpreted as a convergent descriptive comparison rather than independent validation. PVS differs from MELD by integrating additional laboratory abnormalities into a longitudinal fusion score. The present pilot study was not designed to establish superiority over MELD or individual laboratory parameters; direct comparative prognostic evaluation will require an independent clinical endpoint and larger cohorts.

### 4.2 Amanitin intoxication as a severe event

In amatoxin poisoning, the modular PVS application demonstrated potential utility as this severe condition affects previously healthy patients, often showing a sharp increase in severity followed by rapid remission. Some severely affected patients had ALT levels above 10,000 U/L and AST levels up to 8,776 U/L, indicating severe liver damage.

The typical clinical course of amatoxin poisoning begins with gastrointestinal symptoms such as vomiting and diarrhea, often followed by transient improvement before progressive hepatocellular injury. In severe cases, impaired hepatic synthesis can lead to coagulopathy and hepatic encephalopathy, and renal injury may also occur. This course reflects amatoxin-mediated inhibition of RNA polymerase II and subsequent disruption of protein synthesis. Management includes early supportive treatment, consideration of silibinin and N-acetylcysteine, and early assessment at a liver transplant center in severe cases (Caré et al., 2024; Tan et al., 2022). Recent multicenter data have also evaluated therapeutic plasma exchange in amatoxin-associated acute liver failure (Stahl et al., 2025). Treatment may modify laboratory trajectories and therefore the PVS course. Detailed patient-level timing of antidotal and supportive treatments was not available in the laboratory-focused dataset, so changes in PVS cannot be attributed to spontaneous disease evolution *versus* treatment effects. One patient underwent liver transplantation, which is reported descriptively as a severe clinical outcome. The available dataset did not permit determination of whether PVS_last_ was measured before or after liver transplantation.

Understanding disease progression is critical. Among patients with a matched ingestion time, the median time to peak PVS was 3.03 days after ingestion (Q1–Q3, 2.13–3.54; n = 15), followed by declining PVS values in many patients. These trajectories provide a descriptive time frame for laboratory severity evolution after ingestion. Outcomes varied widely, ranging from death or liver transplantation to minimal or no symptoms.

This study also examined the relationship between urinary Amanitin levels and disease severity and found no significant relationship with the highest PVS, PVS near the time of measurement, or Amanitin concentration relative to the time of ingestion. Of note, the deceased patient had only 5.1 μg/L, and the liver transplant patient had 6 μg/L. In this small cohort, we found no evidence of an association between urinary Amanitin concentration and the evaluated severity measures. The limited sample size and variable sampling times constrain the interpretation of this negative finding.

### 4.3 Prospects and limits of modular severity assessment

This study evaluated the PVS against other variables, demonstrating its adaptability to different measurement frequencies and time frames. This flexibility allows multivariate correlations to be condensed into a single score, useful across medical specialties. In laboratory medicine, it facilitates the quantification of disease severity, providing a compact longitudinal summary of disease severity.

This pilot study analyzed retrospective internal data and laid the foundation for PVS applications in novel contexts. Multicenter studies are needed for further validation and refinement before clinical use. Currently, PVS is a customizable tool for specific scenarios instead of a universal scoring system. While adaptable, its current retrospective nature limits its applicability at the bedside. The primary goal remains to transform PVS into a MELD-like score for rapid clinical calculation using established reference values. Future studies would benefit from sensitivity analysis of PVS variables. Optimizing mathematically robust variables while minimizing less influential variables could refine accuracy and applicability. This adaptability supports further evaluation of PVS as a tool for integrating complex clinical data. Figure 1 illustrates its versatility in synthesizing patient data at various intervals and frequencies.

## 5 Conclusion

By integrating common laboratory values, this study demonstrates the feasibility of adapting PVS for laboratory-based severity assessment in Amanitin-intoxicated patients. The resulting score provides a longitudinal summary of laboratory abnormalities, particularly during acute hepatic injury. Clustering patients based on PVS scores further described between-patient differences in laboratory severity profiles. No significant association was found between urinary Amanitin levels and disease severity in this cohort; the small sample and variable sampling times limit interpretation of this negative finding.

While these findings are promising, further validation through multicenter and prospective studies is essential to confirm the utility of the PVS in clinical practice. The modular nature of the PVS makes it a flexible, scalable tool with potential applications beyond Amanitin poisoning, including other severe poisonings and critical care settings.

## Data Availability

The data analyzed in this study is subject to the following licenses/restrictions: The analysis code is available at https://github.com/phohland/PVS. Requests to access these datasets should be directed to the corresponding author.
