# Supplementary material for: Quantifying disease severity in amanitin-intoxicated patients by fusing common laboratory values
Source: Front Toxicol. 2026 Oct 14;8:1995921. doi: 10.3389/ftox.2026.1995921 (PMC13713024; doi:10.3389/ftox.2026.1995921)
Supplement: Supplementary file 1 [file Supplementaryfile1.docx]

Supplemental Material

# 1 Normal and reference values used for PVS scaling

| **Variable** | **Normal value** | **Reference raw value** | **Reference deviation** |
| --- | --- | --- | --- |
| Quick | 100 % | 11.9 % | 88.1 % |
| ALT | 40 U/L | 5383 U/L | 13357.5 % |
| AST | 20 U/L | 3584 U/L | 17820 % |
| Bilirubin | 10 µmol/L | 199 µmol/L | 1890 % |
| LDH | 200 U/L | 2653 U/L | 1226.5 % |

The normal values represent the predefined clinical laboratory reference values used for PVS calculation. The reference raw values were derived from the five patients in the intermediate-care reference group and represent the measurements producing the largest absolute deviation from the corresponding normal value. The reference deviation expresses this difference relative to the normalized value of 100%. These values were used for the variable-specific scaling of the PVS as described in Section 2.2.

# 2 Descriptive raw data statistics of all intoxicated patients

| Variable | Mean | Median | Min | Max |
| --- | --- | --- | --- | --- |
| Prothrombin time (Quick) | 79.2 % | 90.3 % | 7 % | 126 % |
| ALT | 948 U/L | 177 U/L | 5 U/L | 10373 U/L |
| AST | 634 U/L | 58 U/L | 12 U/L | 8776 U/L |
| Bilirubin | 43.8 µmol/L | 21 µmol/L | 2 µmol/L | 265 µmol/L |
| LDH | 705 U/L | 256 U/L | 151 U/L | 7342 U/L |

**3	Severity Clustering of Intoxicated Patients Based on Maximum PVS**

K-means clustering (k = 4) was performed on patient-level maximum PVS values in the combined dataset. For presentation, the figure shows the 16 intoxicated patients with their assigned clusters, ordered by maximum PVS. The four cluster labels (low, mild, severe, and very severe) are descriptive and are not validated clinical severity classes. The cluster centroids in the combined clustering dataset were 0.14, 0.78, 1.05, and 1.53. The horizontal dotted line marks PVS = 1, the internally defined reference scaling level rather than a validated clinical decision threshold.

**4	Longitudinal mixed-effects model reporting and diagnostics**

Full fixed-effect estimates and model-level variance components for the five retained longitudinal mixed-effects models are reported below. Variables entered the component-level models in standardized form. Residual diagnostics were assessed using residual-versus-fitted and normal Q-Q plots, with additional screening of standardized residuals.

**Fixed-effect estimates of the longitudinal mixed-effects models.**

| Model | Term | Estimate | SE | 95% CI | df | p |
| --- | --- | --- | --- | --- | --- | --- |
| Quick | Intercept | 0.090 | 0.091 | -0.089 to 0.268 | 25.19 | 0.334 |
| Quick | Quick | -0.752 | 0.040 | -0.831 to -0.674 | 352.34 | <0.001 |
| Quick | Time | 0.181 | 0.094 | -0.003 to 0.365 | 57.71 | 0.059 |
| Quick | Quick x time | -0.372 | 0.081 | -0.531 to -0.212 | 360.56 | <0.001 |
| ALT | Intercept | 0.015 | 0.061 | -0.105 to 0.134 | 17.04 | 0.815 |
| ALT | ALT | 1.163 | 0.062 | 1.041 to 1.286 | 151.62 | <0.001 |
| ALT | Time | -0.381 | 0.085 | -0.547 to -0.214 | 58.30 | <0.001 |
| ALT | ALT x time | -0.687 | 0.185 | -1.050 to -0.325 | 107.65 | <0.001 |
| AST | Intercept | 0.047 | 0.107 | -0.163 to 0.258 | 15.15 | 0.668 |
| AST | AST | 0.760 | 0.058 | 0.646 to 0.874 | 451.92 | <0.001 |
| AST | Time | -0.301 | 0.088 | -0.473 to -0.128 | 183.92 | <0.001 |
| AST | AST x time | 0.044 | 0.198 | -0.344 to 0.431 | 453.05 | 0.825 |
| Bilirubin | Intercept | 0.180 | 0.154 | -0.122 to 0.482 | 28.64 | 0.252 |
| Bilirubin | Bilirubin | 0.548 | 0.074 | 0.403 to 0.694 | 349.83 | <0.001 |
| Bilirubin | Time | 0.005 | 0.187 | -0.362 to 0.372 | 105.68 | 0.977 |
| Bilirubin | Bilirubin x time | 0.046 | 0.266 | -0.475 to 0.568 | 344.41 | 0.862 |
| LDH | Intercept | 0.113 | 0.131 | -0.144 to 0.370 | 11.18 | 0.406 |
| LDH | LDH | 0.698 | 0.097 | 0.507 to 0.889 | 241.61 | <0.001 |
| LDH | Time | -0.141 | 0.150 | -0.435 to 0.153 | 92.28 | 0.351 |
| LDH | LDH x time | 0.613 | 0.343 | -0.060 to 1.285 | 241.03 | 0.076 |

*Estimates refer to standardized variables in the component-level models. SE, standard error; CI, confidence interval; df, Satterthwaite degrees of freedom.*

**Model-level variance components and residual screening.**

| Model | Observations | Patients | Random-intercept variance | Residual variance | Max \|standardized residual\| | n with \|residual\| > 3 |
| --- | --- | --- | --- | --- | --- | --- |
| Quick | 402 | 28 | 0.175 | 0.327 | 6.31 | 7 |
| ALT | 457 | 28 | 0.066 | 0.167 | 5.36 | 13 |
| AST | 461 | 28 | 0.300 | 0.090 | 7.24 | 8 |
| Bilirubin | 363 | 26 | 0.497 | 0.426 | 6.28 | 9 |
| LDH | 254 | 27 | 0.400 | 0.134 | 5.94 | 5 |

Standardized residuals with absolute values > 3 were observed in all five models. Visual inspection of the diagnostic plots showed departures from ideal normality and constant residual spread, particularly in the distribution tails. These diagnostics should be considered when interpreting the component-level models descriptively.

**Residual diagnostics for the longitudinal mixed-effects models.**

Residual-versus-fitted and normal Q-Q plots are shown for Quick, ALT, AST, bilirubin, and LDH.

**5	Biochemical time course after ingestion**

Time-course analyses were restricted to the 15 intoxicated patients with a matched ingestion time. For this analysis, the first abnormal value was operationally defined as the first observed measurement crossing the predefined PVS normal value in the clinically adverse direction. It does not necessarily represent the first value outside an individual patient's laboratory reference interval or the biological onset of abnormality.

| Variable | First abnormal n | First abnormal, median (Q1–Q3), days | Peak/nadir n | Peak/nadir, median (Q1–Q3), days |
| --- | --- | --- | --- | --- |
| Quick | 15 | 2.10 (1.96–2.67) | 15 | 3.03 (2.35–3.58), nadir |
| ALT | 15 | 2.31 (2.00–2.70) | 15 | 3.03 (2.70–3.69), peak |
| AST | 15 | 2.07 (1.93–2.48) | 15 | 3.44 (2.87–4.76), peak |
| Bilirubin | 15 | 2.31 (1.96–2.70) | 15 | 4.44 (3.59–7.50), peak |
| LDH | 14 | 2.05 (1.92–2.64) | 15 | 3.03 (2.66–3.54), peak |

Time from ingestion to the first observed abnormal measurement and to the respective nadir or peak. Values are median (Q1–Q3) in days. Predefined PVS normal values were Quick 100%, ALT 40 U/L, AST 20 U/L, bilirubin 10 µmol/L, and LDH 200 U/L. LDH crossed the predefined abnormal threshold in 14 of the 15 patients with a matched ingestion time.

**Individual laboratory trajectories after ingestion.**

Each line represents one intoxicated patient with a matched ingestion time (n = 15). Time is shown in days since ingestion. Panels display ALT (U/L), AST (U/L), bilirubin (µmol/L), LDH (U/L), and Quick (%). Lines connect available measurements for visualization; no values were imputed.

**6	One-sided Quick sensitivity analysis**

A sensitivity analysis was performed in which Quick values at or above 100% contributed no Quick-related deviation, while values below 100% retained the original Quick scaling. All other PVS components and the reference scaling were unchanged.

| Outcome | Scoring | Intoxicated, mean ± SD | Control, mean ± SD | Welch p | Cohen's d |
| --- | --- | --- | --- | --- | --- |
| PVS_max_ | Original two-sided Quick | 0.75 ± 0.54 | 0.12 ± 0.04 | <0.001 | 1.64 |
| PVS_max_ | One-sided Quick | 0.73 ± 0.55 | 0.07 ± 0.05 | <0.001 | 1.70 |
| PVS_mean_ | Original two-sided Quick | 0.32 ± 0.25 | 0.05 ± 0.02 | <0.001 | 1.53 |
| PVS_mean_ | One-sided Quick | 0.32 ± 0.26 | 0.03 ± 0.02 | <0.001 | 1.61 |

Sensitivity analysis comparing the original two-sided Quick scoring with one-sided Quick scoring. Values are mean ± standard deviation. In the one-sided analysis, Quick values at or above 100% contributed no Quick-related deviation. Between-group comparisons used Welch's t-test.
